# Supplementary material for: Maternal Pre-Pregnancy BMI, Offspring Adiposity in Late Childhood, and Age of Weaning: A Causal Mediation Analysis
Source: Nutrients. 2023 Jun 29;15(13):2970. doi: 10.3390/nu15132970 (PMC10343873; doi:10.3390/nu15132970)
Supplement: Supplementary file 1 [file nutrients-15-02970-s001.zip › nutrients-2468270-supplementary.pdf]

Supplementary

Table S1. Characteristics of participants included and not included in the sample
Table S2. Linear regression models on association between mother's weight status and offspring adiposity at 17 years
Table S3. Linear regression models on association between age of weaning and offspring adiposity at 17 years
Table S4. Multinomial logistic regression models on association between maternal weight status and age of weaning
Table S5. Causal mediation effects of age of weaning on the association between maternal weight status and offspring' fat mass index at 17 years
Table S6. Sensitivity analyses of the influence of uncontrolled mediator-outcome confounding
Supplementary methods section
Figure S1. DAG showing how the unmeasured confounder U confounds the mediator-outcome association

Table S1. Characteristics of participants included and not included in the sample

		Excluded	Included	Total
Categorical variables	N (%)	9759 (66.5)	4920 (33.5)	14679
Mother weight status	Underweight	369(5.72)	201(4.09)	570(5.01)
	Normal	4703(72.95)	3755(76.32)	8458(74.41)
	Overweight	993(15.40)	719(14.61)	1712(15.06)
	Obesity	382(5.93)	245(4.98)	627(5.52)
	Total	6447	4920	11367
Mother education	CSE	1930(26.12)	540(11.13)	2470(20.18)
	Vocational	857(11.60)	353(7.28)	1210(9.89)
	O level	2552(34.54)	1680(34.63)	4232(34.58)
	A level	1392(18.84)	1360(28.04)	2752(22.49)
	Degree	657(8.89)	918(18.92)	1575(12.87)
	Total	7388(100)	4851(100)	12239(100)
Mother smoking during pregnancy	Never	3513(44.14)	2828(58.31)	6341(49.51)
	Stopped smoking during pregnancy	2424(30.46)	1392(28.70)	3816(29.79)
	Smoked during pregnancy	2021(25.40)	630(12.99)	2651(20.70)
	Total	7958(100)	4850(100)	12808(100)
Mother drinking during pregnancy	Never	3718(46.79)	2108(43.24)	5826(45.44)
	<1 glass per week	2947(37.09)	2017(41.37)	4964(38.72)
	>=1 glass per week	1281(16.12)	750(15.38)	2031(15.84)
	Total	7946(100)	4875(100)	12821(100)
Offspring gender	Male	5284(54.14)	2202(44.76)	7486(51.00)
	Female	4475(45.86)	2718(55.24)	7193(49.00)
	Total	9759(100)	4920(100)	14679(100)
Breastfeeding	Yes still	1437(22.86)	1787(36.52)	3224(28.84)
	Yes Stopped	2931(46.63)	2314(47.29)	5245(46.92)
	Never	1917(30.50)	792(16.19)	2709(24.24)
	Total	6285(100)	4893(100)	11178(100)
Formula feeding	Never	1166(18.21)	1018(20.69)	2184(19.29)
	Yes	5236(81.79)	3902(79.31)	9138(80.71)
	Total	6402(100)	4920(100)	11322(100)
Age of weaning	>=3month	5096(81.31)	4288(87.15)	9384(83.88)
	<3month	1171(18.69)	632(12.85)	1803(16.12)
	Total	6267(100)	4920(100)	11187(100)
**Continuous variables**		**mean (SD)**	**mean (SD)**	
Mother's age		27.6 (4.9)	29.4 (4.5)	
n		6865	4791	
Mother BMI(kg/m^2)		22.99 (4.0)	22.85 (3.6)	

n	6447	4920
Father BMI(kg/m^2)	25.3(3.4)	25.0(3.2)
n	4478	3734
Child birth weight(kg)	3.4(0.6)	3.4(0.5)
n	8743	4858
Offspring BMI at age 17(kg/m^2)	23.3 (4.6)	22.6 (4.1)
n	1160	4920
Age of weaning(weeks)	3.0(1.0)	3.2(0.9)
n	6267	4290

Table S2. Linear regression models on association between mother's weight status and offspring adiposity at 17 years

Maternal weight	Offspring BMI (kg/m^2)				Offspring FMI (kg/m^2)			
	Model0		Model1		Model0		Model1	
	β	95%CI	β	95%CI	β	95%CI	β	95%CI
Normal weight	ref		ref		ref		ref	
OWOB	2.77	2.50 to 3.05	2.67	2.39 to 2.96	2.34	2.05 to 2.63	2.32	2.06 to 2.57

Linear regression model. Model0: unadjusted model. Model1 was adjusted for maternal education, smoking, drinking, age, and offspring sex

BMI, body mass index; FMI, fat mass index; OWOB, overweight or obesity; CI, confidence interval

Table S3. Linear regression models on association between age of weaning and offspring adiposity at 17 years

	Offspring BMI (kg/m^2)					
	Model0		Model1		Model2	
Age of weaning	β	95%CI	β	95%CI	β	95%CI
>3month	ref		ref		ref	
at 3month	0.26	0.01 to 0.52	0.11	-0.16 to 0.37	0.08	-0.19 to 0.34
<3month	1.03	0.65 to 1.41	0.63	0.24 to 1.03	0.58	0.18 to 0.98
	Offspring FMI (kg/m^2)					
Age of weaning	β	95%CI	β	95%CI	β	95%CI
>3month	ref		ref		ref	
at 3month	0.08	-0.19 to 0.34	0.08	-0.16 to 0.31	0.06	-0.18 to 0.30
<3month	0.44	0.04 to 0.83	0.32	-0.04 to 0.68	0.28	-0.08 to 0.64

Model0: unadjusted

Model1: adjusted for maternal education, smoking, drinking, age, child gender

Model2: model1 + adjustment forbreastfeeding and formula

BMI, body mass index; FMI, fat mass index; CI, confidence interval

Table S4. Multinomial logistic regression models on association between maternal weight status and age of weaning

	Model 0		Model 1[a]	
	RRR	95% CI	RRR	95% CI
Age of weaning<3m				
Maternal OWOB	1.58	1.26 to1.98	1.40	1.10 to 1.78
Age of weaning=3m				
Maternal OWOB	1.16	0.99 to 1.37	1.13	0.95 to 1.34
Age of weaning >3m	Base outcome			

RRR, ratio of relative risk; CI, confidence interval

[a]Model1: adjusted for maternal education, smoking, drinking, age, child sex

Table S5. Causal mediation effects of age of weaning on the association between maternal weight status and offspring' fat mass index at 17 years

Estimate	G-computation estimate (MD)	95%CI	Bootstrap Std. Err.	z	P value
TCE	2.28	1.96 to 2.61	0.17	13.83	0.00
NDE	2.28	1.95 to 2.60	0.17	13.75	0.00
NIE	0.01	-0.007 to 0.022	0.01	1.01	0.31
PM	0.00	-0.003 to 0.010	0.00	1.00	0.32
CDE[a]	2.28	1.95 to 2.60	0.17	13.76	0.00

Mediator was models with mlogit regression model, offspring's fat mass index was with linear regression model. NIE are expressed as the expected difference in offspring's FMI when weaning age takes the value observed in individuals with mothers who were overweight or obese compared to those with normal weight. PM is estimated as NIE/TCE. Monte Carlo simulations were used to obtain the CI.

TCE, total causal effect; NDE, natural direct effect; NIE, natural indirect effect; PM, proportion mediated; CDE, controlled direct effect; FMI, fat mass index.
[a]CDE, controlled at >3month

Supplementary methods section

We conducted sensitivity analyses to assess the extent to which the lack of adjustment for U might change the direct and indirect effect estimates. Suppose U is an unmeasured confounding for the mediator-outcome relationship, i.e. it affects both the weaning age and offspring's BMI. Examples of such a U could be infant sleeping quality, family support, etc.

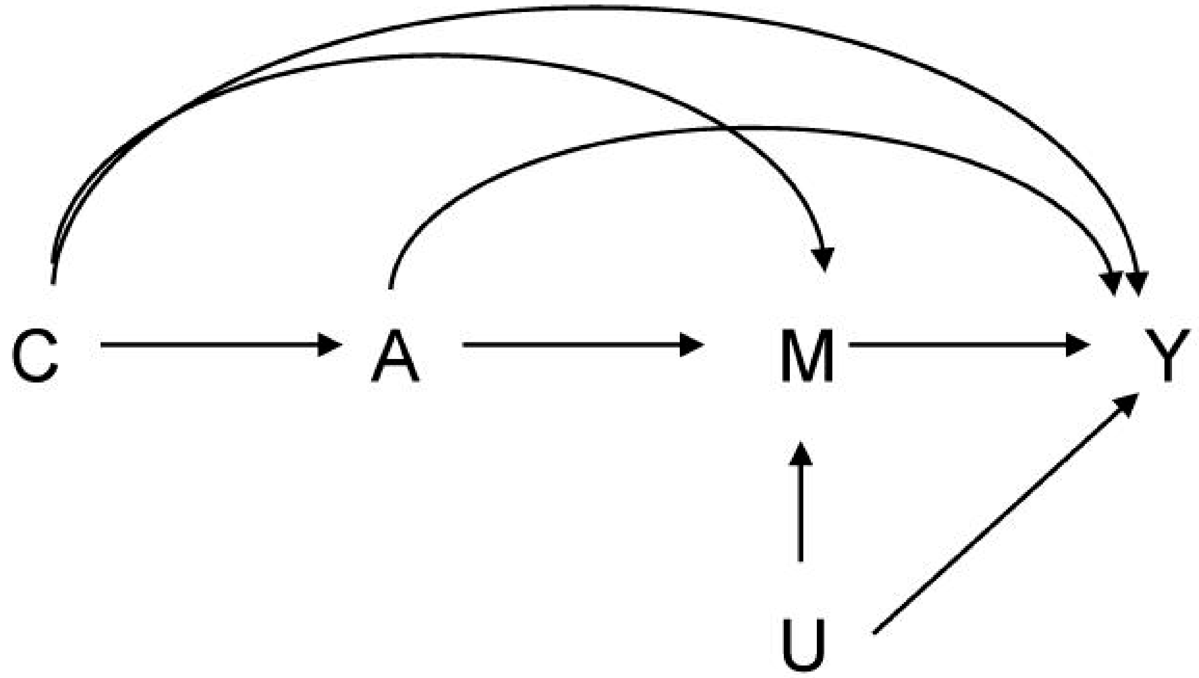

Figure S1. DAG showing how the unmeasured confounder U confounds the mediator-outcome association

A: exposure (maternal OWOB and normal weight)
Y: outcome (offspring's BMI)
M: mediator (weaning age)
C: confounders for exposure-outcome and exposure-mediator relationship, we controlled for maternal education, age, smoking, and drinking before pregnancy
U: unmeasured confounder for mediator-outcome relationship

Here we applied VanderWeele's approach to assess the sensitivity analysis of direct effect and indirect effect[1].

Suppose further that U is binary, that $E[Y|a, m, c, U = 1] - E[Y|a, m, c, U = 0]$ is constant across strata of a, m, c so that $E[Y|a, m, c, U = 1] - E[Y|a, m, c, U = 0] = \gamma$ and that $P(U = 1|a, m, c) - P(U = 1|a^*, m, c)$ is constant across strata of c and m so that $P(U = 1|a, m, c) - P(U = 1|a^*, m, c) = \delta$; then

$$Bias(NDE_{a,a^*}(a^*)) = \gamma\delta$$
$$Bias(NIE_{a,a^*}(a)) = -\gamma\delta$$

We assume individuals with the presence of U had on average 0.2 unit higher BMI (kg/m^2); that is,

$$\gamma = E[Y|a, m, c, U = 1] - E[Y|a, m, c, U = 0] = 0.2$$

for all values of a, m and c.

Conditional on weaning age and the confounders, mothers who were OWOB had 0.2 higher probability of having the U presence.

$$\delta = P(U = 1|a, m, c) - P(U = 1|a^*, m, c) = 0.2$$

for all values of m and c, where a = OWOB and a* = Normal weight.

Under these assumptions, the bias is

$$Bias(NDE_{a,a^*}(m)) = \delta\gamma = 0.04$$
$$Bias(NIE_{a,a^*}(m)) = -\delta\gamma = -0.04$$

We also considered scenarios where *$\delta = 0.3$ and $\delta = 0.4$, respectively.* The corrected estimates for the NDE and the NIE are listed below:

Table S6. Sensitivity analyses of the influence of uncontrolled mediator-outcome confounding

Estimate	Original results		Mild unmeasured confounding ($\delta = 0.2$)		Moderate unmeasured confounding ($\delta = 0.3$)		Strong unmeasured confounding ($\delta = 0.4$)	
	Effect	95%CI	Effect	95%CI	Effect	95%CI	Effect	95%CI
NDE	2.63	2.27 to 2.99	2.59	2.23 to 2.95	2.57	2.21 to 2.93	2.55	2.19 to 2.91
NIE	0.02	0.003 to 0.042	0.06	0.043 to 0.063	0.08	0.063 to 0.102	0.1	0.083 to 0.122

NDE, natural direct effect; NIE, natural indirect effect; BMI, body mass index; CI, confidence interval
U denotes unmeasured confounding between mediator-outcome, that affecting both the age of weaning and offspring's BMI.

If there is unmeasured confounding between mediator-outcome relationship, the NDE might be overestimated and NIE be underestimated. We found that even under the worst scenario (strong unmeasured confounding), the estimated NIE is 0.1 (95%CI: 0.08 to 0.12), which corresponds to 3.7% of the total effect. The majority of the effect is still due to the direct effect of maternal weight status. The conclusion remains similar.

Reference

1. VanderWeele TJ. Bias formulas for sensitivity analysis for direct and indirect effects. *Epidemiology (Cambridge, Mass).* 2010;21(4):540.
